# Differential Gene Expression in Human Fibroblasts Simultaneously Exposed to Ionizing Radiation and Simulated Microgravity

**DOI:** 10.3390/biom14010088

**Published:** 2024-01-10

**Authors:** Polina Malatesta, Konstantinos Kyriakidis, Megumi Hada, Hiroko Ikeda, Akihisa Takahashi, Premkumar B. Saganti, Alexandros G. Georgakilas, Ioannis Michalopoulos

**Affiliations:** 1 Center of Systems Biology, Biomedical Research Foundation of the Academy of Athens, 11527 Athens, Greece; ge18814@mail.ntua.gr (P.M.); kkyriaki@ucsc.edu (K.K.); 2 DNA Damage Laboratory, Physics Department, School of Applied Mathematical and Physical Sciences, National Technical University of Athens, 15780 Athens, Greece; 3 Laboratory of Pharmacology, School of Pharmacy, Aristotle University of Thessaloniki, 54124 Thessaloniki, Greece; 4 UC Santa Cruz Genomics Institute, Santa Cruz, CA 95060, USA; 5 Radiation Institute for Science & Engineering, Prairie View A&M University, Prairie View, TX 77446, USA; mehada@pvamu.edu (M.H.); pbsaganti@pvamu.edu (P.B.S.); 6 Department of Life Science, Faculty of Science and Engineering, Kindai University, Higashiosaka 577-8502, Japan; hi-ikeda@life.kindai.ac.jp; 7 Gunma University Heavy Ion Medical Center, Maebashi 371-8511, Japan; a-takahashi@gunma-u.ac.jp

**Keywords:** space flight, cosmic radiation, microgravity, differentially expressed genes, gene networks

## Abstract

During future space missions, astronauts will be exposed to cosmic radiation and microgravity (μG), which are known to be health risk factors. To examine the differentially expressed genes (DEG) and their prevalent biological processes and pathways as a response to these two risk factors simultaneously, 1BR-hTERT human fibroblast cells were cultured under 1 gravity (1G) or simulated μG for 48 h in total and collected at 0 (sham irradiated), 3 or 24 h after 1 Gy of X-ray or Carbon-ion (C-ion) irradiation. A three-dimensional clinostat was used for the simulation of μG and the simultaneous radiation exposure of the samples. The RNA-seq method was used to produce lists of differentially expressed genes between different environmental conditions. Over-representation analyses were performed and the enriched biological pathways and targeting transcription factors were identified. Comparing sham-irradiated cells under simulated μG and 1G conditions, terms related to response to oxygen levels and muscle contraction were identified. After irradiation with X-rays or C-ions under 1G, identified DEGs were found to be involved in DNA damage repair, signal transduction by p53 class mediator, cell cycle arrest and apoptosis pathways. The same enriched pathways emerged when cells were irradiated under simulated μG condition. Nevertheless, the combined effect attenuated the transcriptional response to irradiation which may pose a subtle risk in space flights.

## 1. Introduction

Space flight conditions differ to those on Earth due to cosmic ionizing radiation (IR) and the absence of gravity, known as microgravity (μG), both of which pose health risk factors to humans, causing complex DNA damage and genome instability [1,2,3,4,5]. It is crucial to gain more insights on these factors, as astronauts will be continuously exposed to them in long-duration exploration missions, such as those to the Moon or Mars, which require humans to remain in space for days, months and even years.

Space radiation risks, arising mainly from solar energetic particles (SEPs) and galactic cosmic rays (GCRs), involve a flux comprising 2% electrons and 98% nuclei, with the nuclear component being ~87% hydrogen, ~12% helium and ~1% high atomic number and energy (HZE) particles [6]. Despite low GCR particle flux levels, their high linear energy transfer (LET) induces intense ionization in matter [7,8]. During Earth-to-Mars manned missions using Hohmann transfer [9] which will last 2–3 years [10], astronauts will face an estimated ~1.01 Sv [11] radiation exposure, increasing the risk of cancer, central nerve system decrements, degenerative tissue effects [12], and irreversible chromosomal instability risks due to HZE particle exposure.

During lengthy planetary flights, astronauts face both potential radiation hazards and simultaneous exposure to microgravity (μG). Living organisms undergo physiological changes in varying gravitational conditions, including muscle atrophy, reduced bone density, immune function decline and endocrine disorders. The space environment, comprising GCRs and reduced gravity, necessitates testing for possible additive or synergistic effects. Altered DNA repair mechanisms due to gravity changes can impede cellular responses to space radiation, increasing the risk of DNA damage accumulation and tumorigenesis [13]. Recent studies highlight that spaceflight stressors like ionizing radiation (IR) and/or microgravity disrupt the wound healing process, affecting pathways like inflammation and proliferation [14]. Microgravity significantly impacts cell death, migration and gene expression in tumor cells, including cancer stem cells, and alters the effects of chemotherapeutic drugs [15]. While extensive research has focused on the impact of either radiation or microgravity alone, limited studies have addressed their combined effects. Previous attempts were found to be challenging, as older clinostats had to pause rotation which simulates microgravity to irradiate the cells [16,17], potentially introducing additional gravitational stimuli, and thus activating specific signaling cascades.

In deep space beyond the Van Allen belts, galactic cosmic rays consist of both high-energy and low-energy radiation. To investigate the combined effects of space radiation and microgravity, considering lunar and Mars explorations and long-term stays in space in the near future, it was decided to use carbon ions as high-energy radiation and X-rays as low-energy radiation. To maintain the consistent simulated μG condition before, during and after exposure to radiation, a 3D clinostat that allows samples to be rotated and irradiated simultaneously was developed [18] and used in this study. This clinostat was previously used for the study of the differential expression of exclusively nine cell cycle-related genes in response to X-ray or Carbon-ion (C-ion) irradiation, with and without simulated μG, in human fibroblasts [1]. The raw transcriptomic data produced from that previous study [1] were reanalyzed in the current work, applying a systems biology approach, to identify all differentially expressed genes between various conditions and their predominant processes they participate in and to identify a possible synergy between radiation and μG.

## 2. Materials and Methods

1BR-hTERT human fibroblast cells were cultured in CO_2_-independent medium (Thermo Fisher Scientific, Waltham, MA, USA) supplemented with 10% (*v*/*v*) fetal bovine serum (MP Biomedicals, Santa Ana, CA, USA), 200 mM L-glutamine (Thermo Fisher Scientific, Waltham, MA, USA), and penicillin–streptomycin mixed solution (Nacalai Tesque, Kyoto, Kyoto, Japan) at 37 °C under 1G or simulated μG for 48 h in total. The samples were collected 0 (sham irradiated), 3 or 24 h after X-ray or C-ion irradiation at 1 Gy. X-ray irradiation was performed using an X-ray generator (200 kV, 14.6 mA, aluminum filter (0.3 mm thick), MultiRad225, Faxitron Bioptics, LLC, Tucson, AZ, USA) equipped with a high-speed shutter. C-ion irradiation was performed using a synchrotron (Gunma University Heavy Ion Medical Center (GHMC), Maebashi, Gunma, Japan) and respiratory gating signals with a dose-averaged LET of 50 keV/μm at the center of the 6 cm spread-out Bragg peak of the beam with energy of 290 MeV/n. The dose rate was approximately 0.03 Gy/min for both X-ray and C-ion irradiation under the simulated μG or 1G conditions. Simulated μG was accomplished using a three-dimensional clinostat [18,19]. This device can manipulate the effect of gravity through the 3D rotation of two orthogonal axes and by continuously changing the direction of gravity. The X:Y ratios of clino-rotation were set at 11:13 rpm and 66°/s:78°/s using a special controller to maintain suitable conditions, which means that it does not use random speed and random direction. The rotation angle between the *Z*-axis of the 3D clinostat (i.e., the axis of radiation exposure) and the normal line of the sample holder on the 3D clinostat, θ, was kept at less than 12°, assuming the X:Y ratio of the clino-rotation was 11:13 rpm. Because different research groups are performing simulated microgravity experiments under various conditions with different types of simulators and cell line, we think that it is important to carefully consider the experimental conditions and provide details such as simulator limitations in order to avoid misinterpretation of the results [20]. Among such limitations, we used adherent human fibroblasts in a thin culture vessel completely filled with medium (without bubbles) to minimize stress on the cells as much as possible. It is not necessary to change the medium under our simulated microgravity conditions until the sampling. From our previous data of cell growth, which did not differ significantly between rotating and standing conditions after 48 h of culture, we believe that it is unlikely that cells are subjected to shear stress under our experimental conditions using our system. The samples were irradiated when in horizontal position, without pausing the rotation, for 0.2 s every minute. In total, 12 conditions were studied (Table 1) in triplicate [1].

### 2.1. RNA-Sequencing

RNA from each of the 36 samples, was extracted using TRIzol™ Reagent (Thermo Fisher Scientific, Waltham, MA, USA) and its quality was assessed using the RNA 6000 Pico Kit (Agilent Technologies, Santa Clara, CA, USA). rRNA was depleted using the NEBNext^®^ rRNA Depletion Kit (New England Biolabs, Ipswich, MA, USA). Then, RNA-Seq library was prepared using NEBNext^®^ Ultra™ Directional RNA Library Prep Kit for Illumina^®^ (New England Biolabs, Ipswich, MA, USA). Paired-end sequencing (2 × 36 bp) was performed with NextSeq 500 (Illumina, San Diego, CA, USA) at Tsukuba i-Laboratory LLP (Tsukuba, Ibaraki, Japan) [1]. Four FASTQ files were produced from each sample.

### 2.2. Differentially Expressed Gene Analysis

**Figure 1:**
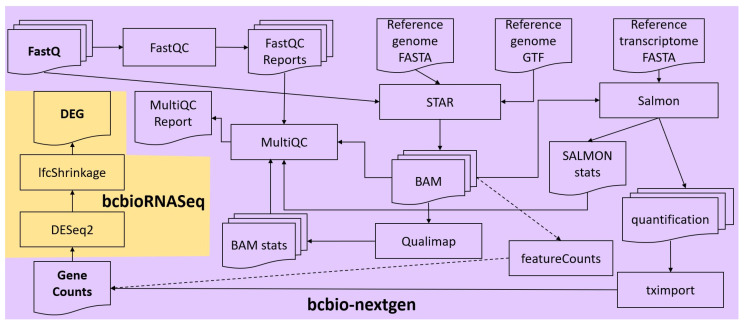
RNA-seq analysis pipeline. Gene counts were produced from FASTQ files, through the bcbio-nextgen pipeline (in lavender background). Lists of differentially expressed genes were produced through the bcbioRNASeq pipeline (in light orange background).

FASTQ files of each sample were concatenated, and the integrity of the resulting files was checked, using in-house scripts. Quality control and alignment of their reads were carried out via the RNA-seq workflow from the bcbio-nextgen bioinformatics framework (version 1.2.9) [21] (Figure 1). To ensure that the library generation and sequencing quality were suitable for further analysis, FastQC [22] was used to examine the raw reads for quality issues. Then, raw reads were aligned to the GRCh38 (hg38) version of the human reference genome (FASTA and GTF files) with the splice-aware aligner STAR (version 2.6.1d) in two-pass mode (the first pass discovers new splice junctions and inserts them into the junction database, and the second pass calls junctions and calculates their counts) [23]. Moreover, Salmon (version 1.7.0) was run in alignment-based mode, using the genome alignments from STAR (BAM files) and the reference transcriptome FASTA file, to generate abundance estimates for known splice isoforms [24]. Bcbio assessed the complexity and quality of the RNA-seq data by quantifying ribosomal RNA (rRNA) content and the genomic context of alignments in known transcripts and introns using a combination of custom tools and Qualimap [25]. MultiQC [26] was then used for quality control and assurance analysis of the resulting BAM files by comparing to metrics gathered from bcbio-nextgen, samtools [27], Salmon, STAR, Qualimap, and FastQC. Next, we quantitated reads by assigning them to genes (features) annotated in Ensembl (release 105) and counting them with the featureCounts tool [28] or preferably tximport [29]. Gene counts were processed for DGEA, using DESeq2 (version 1.38.2) [30] default settings through the bcbioRNASeq R package (version 0.5.1) [31] (Figure 1). Moreover, log fold change shrinkage for visualization and ranking was performed calling the lfcShrink function of the DESeq2 package, replacing *p*-values with s-values produced by the apeglm estimation method [32]. S-value was proposed as a statistic giving the aggregate false sign rate for tests with equal or lower s-value than the one considered [33]. Exported lists containing statistically significant differentially expressed genes (DEGs) include metrics such as Log_2_ Fold Change (Log_2_FC) and s-values for each gene. The lists were further annotated by bcbioRNASeq to include HGNC [34] gene symbols and names. The threshold for statistical significance was set at s-value < 0.001, as suggested [35]. Using this method, the statistically significant differentially expressed genes between various pairs of biological conditions (Table 2) were identified.

### 2.3. Biological Term Enrichment Analysis

Gene term enrichment analyses were performed to identify the prevalent biological processes and pathways over- and under-expressed genes of each DEG analysis participate in using WebGestalt [36]. The over-representation analysis (ORA) method [37] was employed, applying BH [38] multiple test adjustment. The threshold for statistical significance was set at a false discovery rate (FDR) < 0.05. The functional databases that were used for biological term enrichment were Biological Process, Cellular Component and Molecular Function from Gene Ontology [39], KEGG [40] and Transcription Factor Targeting and miRNA Targeting networks from MSigDB [41]. In order to identify and depict overlapping genes or biological terms between comparisons of conditions, Venn diagrams were produced using a webtool developed by the Bioinformatics and Evolutionary Genomics group of Ghent University at https://bioinformatics.psb.ugent.be/webtools/Venn/ (accessed on 7 January 2022).

### 2.4. Protein–Protein Interaction Network Analysis

STRING [42]-based protein–protein interaction (PPI) network analyses were performed for the DEGs and PPI networks were constructed for each DEG analysis from all comparisons in order to discover their functional associations. The average local clustering coefficient [43] served as a measure of how connected the produced PPI networks were and PPI enrichment *p*-values provide the probability to obtain the observed number of edges by chance. To identify the hub genes of the PPI networks, i.e., the genes with the highest degree of connectivity, the interactions of each gene were counted and genes with the highest number of edges were pinpointed.

## 3. Results

Lists of up- and down-regulated genes were produced through the DEG analysis from all comparisons between two different biological conditions and subsequently, biological term over-representation analyses were performed.

### 3.1. Early-Response Genes to X-ray Irradiation under 1G

From the comparison between X-ray (low-LET) irradiated cells collected 3 h post-irradiation and sham-irradiated ones under 1G (X3G-X0G) (Table S1), 112 over-expressed genes were found in total, and among them, *CDKN1A*, *MDM2*, *PURPL*, *PTCHD4*, *TP53INP1*, *PAPPA* and *BTG2* stood out. In particular, the expression of *CDKN1A* and *MDM2* had approximately quadrupled. Likewise, 108 genes were found to be statistically significant under-expressed ones. Down-regulated genes *FAM111B*, *ZNF367* and *MCM10* stood out. Concerning the over-expressed genes, enrichment analyses for Gene Ontology biological processes and KEGG pathways highlighted the p53 signaling pathway. Biological processes related to response to stimulus and apoptosis were also identified. Focusing on under-expressed genes, biological term over-representation analyses in all Gene Ontology aspects, as well as in KEGG pathways, highlighted cell cycle and carcinogenesis-related terms. E2F was identified as a transcription factor targeting down-regulated genes.

### 3.2. Late-Response Genes to X-ray Irradiation under 1G

From the comparison between X-ray-irradiated cells collected 24 h post-irradiation (late response) and sham-irradiated ones under 1G (X24G-X0G) (Table S2), 571 up- and 1026 down-regulated genes were found. The over-expressed genes *PURPL*, *PTCHD4* and *PAPPA* were found to be predominant. *PURPL*, for example, suppresses basal p53 levels and promotes tumorigenicity in colorectal cancer [44]. Enrichment analyses in all Gene Ontology aspects highlighted terms related to cell proliferation and cardiovascular system development, such as angiogenesis. The main KEGG pathway identified was as rather expected, the p53 signaling pathway, indicating the response to varying types of stresses like radiation, hypoxia, oxidative attack [45] and even simulated μG [46]. FOXO4 was identified as the transcription factor controlling the expression of up-regulated genes. Among the down-regulated genes, many stood out. A few were *MKI67*, *ASPM*, *CENPF*, *ANLN*, *CDC20*, *DLGAP5*, *CCNB1*, *CEP55* and *PLK1*. Enrichment analyses in all Gene Ontology domains, as well as in KEGG pathways, highlighted cell cycle and DNA repair-related terms. E2F was found to control the expression of down-regulated genes.

### 3.3. Late- vs. Early-Response Genes to X-ray Irradiation under 1G

From the comparison between X-ray-irradiated cells collected 3 (early) and 24 (late) h post-irradiation under 1G (X24G-X3G) (Table S3), 255 up- and 619 down-regulated genes were identified. For the up-regulated genes, enrichment analyses concerning Gene Ontology aspects highlighted anatomical morphogenesis and response to stimulus related terms. The predominant KEGG pathway identified was mitophagy. FOXO4 was identified as an up-regulated gene-targeting transcription factor. Among the down-regulated genes, *MKI67*, *ASPM*, *CENPF*, *TOP2A* and *PRC1* stood out. Over-representation analyses in all Gene Ontology categories, as well as in KEGG pathways, highlighted cell cycle and DNA repair related terms. E2F was identified as an under-expressed gene-targeting transcription factor.

### 3.4. Early-Response Genes to C-ion Irradiation under 1G

The high LET of C-ions is expected to elicit quantitatively and qualitatively different responses compared to low LET including not only the DNA damage response (DDR) pathways but also inflammatory and immune system activation and systemic effects [47]. From the comparison between C-ion-irradiated cells collected 3 h post-irradiation and sham-irradiated ones under 1G (C3G-C0G) (Table S4), 159 over-expressed genes were identified in total. Among those ones, *CDKN1A*, *MDM2*, *PAPPA*, *TNFRSF10B*, *BTG2*, *TP53INP1*, *PTCHD4* and *PURPL* stood out, in particular, the expression of *CDKN1A* (p21) and *MDM2* had log_2_ fold change of 2.5 and 2.2. *CDKN1A* is a downstream gene to TP53 and often showed to act as a negative regulator of the cellular levels of TP53 [48]. Likewise, 114 genes were found to be under-expressed in a statistically significant manner. The down-regulated genes *PLK1*, *MKI67*, *BUB1B* and *DTL* were most prominent. Concerning the over-expressed genes, enrichment analyses in all Gene Ontology aspects highlighted apoptosis and type I interferon signaling pathway related terms. KEGG pathways identified the p53 signaling pathway. LEF1 was identified as a gene-targeting transcription factor. Concentrating on under-expressed genes, biological term over-representation analyses in all Gene Ontology domains, as well as in KEGG pathways, highlighted cell cycle-related terms. E2F was identified as a targeting transcription factor for under-expressed genes.

### 3.5. Late-Response Genes to C-ion Irradiation under 1G

From the comparison between C-ion irradiated collected 24 h post-irradiation and sham-irradiated cells under 1G (C24G-C0G) (Table S5), 620 up- and 1022 down-regulated genes were found. Over-expressed genes *PTCHD4*, *PURPL*, *BTG2* and *CDKN1A* were found to be predominant. Biological term enrichment analyses in all Gene Ontology categories highlighted terms related to cell proliferation and cardiovascular system development, such as angiogenesis. The predominant KEGG pathway identified was the p53 signaling pathway. The transcription factor Forkhead Box O4 was also identified. Among the down-regulated genes, many stood out. A few were *MKI67*, *ASPM*, *CENPF*, *ANLN*, *CDC20*, *DLGAP5*, *CCNB1*, *CEP55* and *PLK1*. Enrichment analyses in all Gene Ontology aspects, as well as in KEGG pathways, highlighted cell cycle and DNA repair related terms. E2F was identified as a gene-targeting transcription factor.

### 3.6. Late- vs. Early-Response Genes to C-ion Irradiation under 1G

From the comparison between C-ion-irradiated cells collected 24 and 3 h post-irradiation under 1G (C24G-C3G) (Table S6), 147 up- and 563 down-regulated genes were identified. Statistically significant terms were not found after performing over-representation analyses for over-expressed genes. For under-expressed genes, enrichment analyses in all Gene Ontology domains, as well as in KEGG pathways, highlighted cell cycle-related terms. Biological processes related to response to stimulus and DNA repair were identified. E2F was found as a gene-targeting transcription factor. Down-regulated genes, whose expression was found to be expressed 3–5 times less, were *MKI67*, *H2BC18*, *H1-3*, *H2AC18*, *TMPO*, *H4C4* and *H3C3*.

### 3.7. Effects of Simulated μG on Sham-Irradiated Cells

From the comparison of sham-irradiated cells under simulated μG and 1G (X0μG-X0G (Table S7) and C0μG-C0G (Table S8)), up-regulated genes were identified and among those, *PCDHGC4* and *PCLO* were prominent. After performing over-representation analyses for over-expressed genes, statistically significant terms were not found. Down-regulated genes were also identified and *TTN* and *MSTN* were found to be predominant based on their log_2_ fold changes. For under-expressed genes, enrichment analyses in all Gene Ontology aspects highlighted the response to oxygen levels, muscle contraction and regulation of blood circulation-related terms. The prevalent KEGG pathway identified was Pathogenic *Escherichia coli* infection. SRF was identified as a gene-targeting transcription factor for down-regulated genes.

### 3.8. Response to Simulated μG in Cells Collected 3 h after C-ion Irradiation

In the comparison referring to the response to simulated μG in cells collected 3 h after C-ion irradiation (C3μG-C3G) (Table S9), cell cycle-promoting terms, such as cell division, were over-represented in up-regulated genes. In comparisons involving the response to simulated μG in cells collected 3 h after X-ray irradiation (X3μG-X3G) (Table S10), 24 h after X-ray irradiation (X24μG-X24G) (Table S11), or 24 h after C-ion irradiation (C24μG-C24G) (Table S12), no enriched biological terms were identified.

### 3.9. Early-Response Genes to X-ray Irradiation and Simulated μG Combined Effect

From the comparison between X-ray-irradiated cells collected 3 h post-irradiation under simulated μG and sham-irradiated under 1G (X3μG-X0G) (Table S13), 76 over-expressed genes were found and among those *CDKN1A*, *MDM2*, *FDXR*, *PTCHD4*, *TP53INP1*, *BTG2* and *GDF15* stood out. Likewise, 21 under-expressed genes were found to be statistically significant. Down-regulated genes *FAM111B*, *ZNF367* and *VIM-AS1* stood out. Concerning the over-expressed genes, enrichment analyses for Gene Ontology biological processes and KEGG pathways highlighted the p53 signaling pathway. Biological processes related to response to stimulus and apoptosis were also identified. Focusing on under-expressed genes, biological term over-representation analyses highlighted cell cycle-related biological processes.

### 3.10. Late-Response Genes to X-ray Irradiation and Simulated μG Combined Effect

From the comparison between X-ray-irradiated cells collected 24 h post-irradiation under simulated μG and sham-irradiated cells under 1G (X24μG-X0G) (Table S14), 877 up- and 1429 down-regulated genes were found. Over-expressed genes *PTCHD4*, *PURPL* and *PAPPA* were found to be predominant. Enrichment analyses in all Gene Ontology aspects highlighted terms related to cell proliferation and cardiovascular system development. The main KEGG pathway identified was the p53 signaling pathway. The transcription factor FOXO4 was also identified as a trans-activator of up-regulated genes. Furthermore, from over-expressed microRNAs: MIR-17, MIR-20A and MIR-106A were discovered. Among the down-regulated genes, many stood out. A few were *MKI67*, *ASPM*, *TPX2*, *IQGAP3*, *CENPF*, *KIF20B*, *CCNB1* and *CEP55*. Enrichment analyses in all Gene Ontology domains highlighted cell cycle and DNA repair related terms. The predominant KEGG pathway was found to be carcinogenesis. E2F was identified as a gene-targeting transcription factor for under-expressed genes.

### 3.11. Early-Response Genes to C-ion Irradiation and Simulated μG Combined Effect

From the comparison between C-ion-irradiated cells collected 3 h post-irradiation under simulated μG and sham-irradiated under 1G (C3μG-C0G) (Table S15), 184 over-expressed genes were found and among those *CDKN1A*, *MDM2*, *PTCHD4*, *BTG2*, *PAPPA*, *TP53INP1*, and *PURPL* stood out. Likewise, 136 under-expressed genes were found to be statistically significant. Down-regulated genes *MKI67* and *DTL* were found to be prominent. Concerning the over-expressed genes, enrichment analyses in all Gene Ontology aspects, as well as KEGG pathways, highlighted the p53 signaling pathway. LEF1 was identified as a gene-targeting transcription factor for up-regulated genes. Concentrating on under-expressed genes, biological term over-representation analyses in all Gene Ontology domains, as well as KEGG pathway, highlighted cell cycle, circulatory system development and protein digestion and absorption related terms. E2F was identified as a gene-targeting transcription factor.

### 3.12. Late-Response Genes to C-ion Irradiation and Simulated μG Combined Effect

From the comparison between C-ion-irradiated cells collected 24 h post-irradiation under simulated μG and sham-irradiated under 1G (C24μG-C0G) (Table S16), 478 up- and 803 down-regulated genes were found. Over-expressed genes *PTCHD4*, *PURPL*, *BTG2* and *CDKN1A* were found to be predominant. Biological term enrichment analyses in all Gene Ontology categories highlighted terms related to apoptosis and response to stress. The predominant KEGG pathway identified was the p53 signaling pathway. The transcription factor Forkhead Box O4 was also identified. Among the down-regulated genes, many stood out. A few were *MKI67*, *H2BC18*, *H1-3*, *H4C4*, *H3C10*, *H2AC13* and *H3C3*. Enrichment analyses in all Gene Ontology aspects highlighted cell cycle and DNA repair related terms. The predominant KEGG pathways identified are cell cycle and carcinogenesis related. E2F was identified as a gene-targeting transcription factor for under-expressed genes.

### 3.13. Detection of Apoptosis-, DNA Damage Repair- or Cell Cycle-Related Genes

From the comparisons between X-ray or C-ion-irradiated cells collected 3 or 24 h post-irradiation under simulated μG and sham-irradiated under 1G (X3μG-X0G) (Table S13), (X24μG-X0G) (Table S14), (C3μG-C0G) (Table S15) and (C24μG-C0G) (Table S16), apoptosis-related genes *BLOC1S2*, *EDA2R*, *TP53INP1*, *MDM2*, *CDKN1A*, *FAS* and *BCL2L1* were found to be up-regulated, DNA damage repair-related genes *BRCA1*, *POLQ*, *BLM* and *H2AFX* and cell cycle-related genes *MKI67*, *CDT1*, *CDC6*, *MSH6* and *TERT* were found to be down-regulated.

### 3.14. Overlaps between Early- and Late-Response Genes to X-ray and C-ion Radiation under 1G

**Figure 2:**
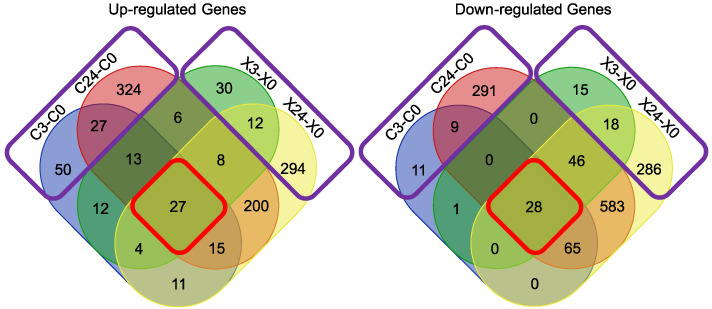
Venn diagrams of up- and down-regulated genes between early- and late-response genes to X-ray and C-ion radiation under 1G. Common up- and down-regulated genes among all four categories are surrounded in red, while common up- and down-regulated genes between early- and late-response genes due to only X-ray or C-ion radiation under 1G are surrounded in purple.

Venn diagrams were created for up- and down-regulated genes between four comparisons under 1G: Early response to C-ion irradiation (C3G-C0G), late response to C-ion irradiation (C24G-C0G), early response to X-ray irradiation (X3G-X0G) and late response to X-ray irradiation (X24G-X0G) (Figure 2). In total, 27 up-(Table 3) and 28 down (Table 4)-regulated genes were found to be overlapping in all four comparisons. Performing over-representation analyses, focusing on the common over-expressed genes, the biological processes highlighted were related to the DNA damage response, signal transduction by p53 class mediator, regulation of cell cycle arrest, intrinsic apoptotic signaling pathway and regulation of catabolic process (regulation of autophagy). For both over- and under-expressed genes, biological processes related to regulation of mitotic cell cycle and DNA integrity checkpoint (G1/S phase transition)-related terms were identified. Furthermore, for under-expressed genes, the biological process of regulation of G2/M transition of mitotic cell cycle was also identified.

### 3.15. Combined Effect of Radiation and Simulated µG on Common Genes Identified between Early- and Late-Response Genes to X-ray and C-ion Radiation under 1G

Among the 27 up (Table 3)- and 28 down-regulated (Table 4) genes between early- and late-response genes to X-ray and C-ion radiation under 1G, 3 up- and 12 down- were found to be statistically significant in at least one of the interactions between early response to X-ray and response to simulated μG ((X3μG-X0μG)-(Χ3G-X0G)) (Table S17), late response to X-ray and response to simulated μG ((X24μG-X0μG)-(Χ24G-X0G)) (Table S18), early response to C-ion and response to simulated μG ((C3μG-C0μG)-(C3G-C0G)) (Table S19) or late response to X-ray and response to simulated μG ((C24μG-C0μG)-(C24G-C0G)) (Table S20). Specifically, for over-expressed genes *TNFRSF10B*, *PTCHD4* and *PURPL* (Table 5) that their expression increased with irradiation alone, simulated μG in combination with irradiation resulted in a lower gene expression increase [1]. Furthermore, it was found that in the 12 genes whose expression was decreased with irradiation alone (Table 6), simulated μG in combination with irradiation resulted in a lower gene expression decrease. None of the aforementioned 27 up- and 28 down-regulated genes were identified in interactions between late vs. early response to X-ray and response to simulated μG (X24μG-X3μG)-(Χ24G-X3G) (Table S21) and in late vs. early response to C-ion and response to simulated μG (C24μG-C3μG)-(C24G-C3G) (Table S22).

### 3.16. High- and Low-LET Radiation

Venn diagrams were created for up- and down-regulated genes (Figure 2) among four comparisons under 1G: early response to C-ion irradiation (C3G-C0G), late response to C-ion irradiation (C24G-C0G), early response to X-ray irradiation (X3G-X0G) and late response to X-ray irradiation (X24G-X0G). C-ion is high-LET (linear energy transfer) radiation, while X-ray is low-LET radiation. Genes that were differentially expressed due to only X-ray or C-ion radiation were identified. It was discovered that 401 up- and 311 down-regulated genes were found due to C-ion radiation only, while 336 up- and 319 down-regulated were due to X-ray. Performing over-representation analyses for over-expressed genes, in the case of C-ion radiation, biological processes related to defense response (immune effector process and type I interferon signaling pathway) and vesicle-mediated transport between Golgi apparatus and endoplasmic reticulum were highlighted, while for X-ray, the biological process of angiogenesis was identified. Focusing on under-expressed genes, in both types of radiation, the biological process of DNA repair was identified. In C-ion radiation, more specific terms were found, such as double-strand break (DSB) repair, DSB repair via non-homologous end joining and non-recombinational repair relating most probably to the inherent repair difficulty of high-LET radiations and the clustering of ionization and induced damage [47]. In X-ray, biological processes related to mitotic cell cycle phase transition were more apparent.

### 3.17. Detection of Oxidase-Related Genes

From the comparisons between X-ray or C-ion-irradiated cells collected 3 or 24 h post-irradiation under 1G (X3G-X0G) (Table S1), (X24G-X0G) (Table S2), (C3G-C0G) (Table S4) and (C24G-C0G) (Table S5), the lysyl oxidase (LOX) genes *LOX*, *LOXL1*, *LOXL2*, *LOXL3* and *LOXL4* were found to be over-expressed as a response to radiation. All three mitochondrially encoded cytochrome c oxidase subunits (*MT-CO1*, *MT-CO2*, *MT-CO3*) and cytochrome c oxidase subunit 7C (COX7C) were also found to be over-expressed. Other oxidases that were discovered to be over-expressed due to IR were Acyl-CoA oxidase 2 (*ACOX2*), Aldehyde oxidase 1 (*AOX1*), Quiescin sulfhydryl oxidase 1 (*QSOX1*) and Glutathione peroxidase 1 (*GPX1*).

### 3.18. Protein–Protein Interaction Networks

**Figure 3:**
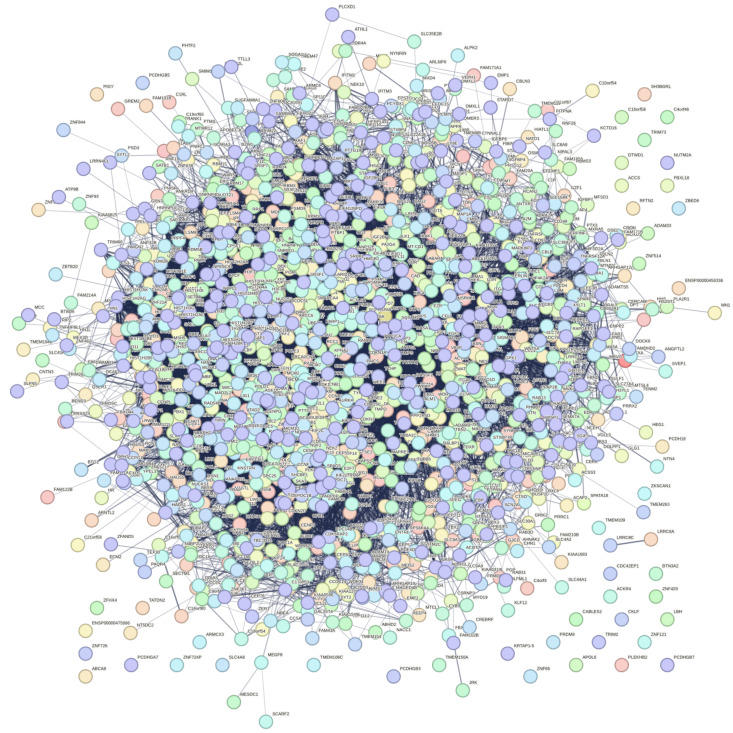
Protein–protein interaction network for DEGs from the comparison between X-ray-irradiated cells collected 24 h post-irradiation and sham-irradiated ones under 1G.

**Figure 4:**
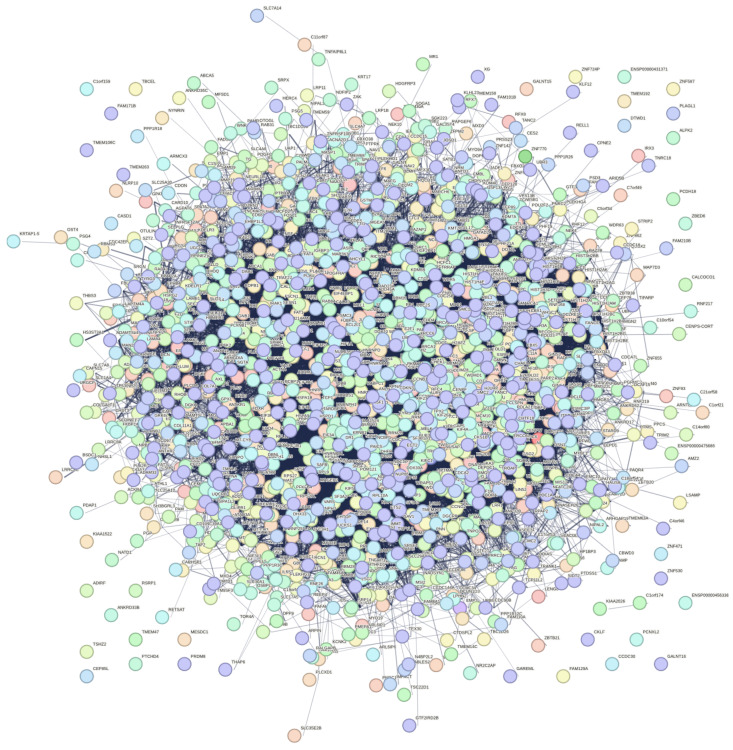
Protein–protein interaction network for DEGs from the comparison between C-ion-irradiated cells collected 24 h post-irradiation and sham-irradiated ones under 1G.

Protein–protein interaction networks were made for all DEGs (both up- and down- regulated genes) from the comparison between X-ray-irradiated cells collected 24 h post-irradiation and sham-irradiated ones under 1G (X24G-X0G) (Figure 3) and between C-ion-irradiated cells collected 24 h post-irradiation and sham-irradiated ones under 1G (C24G-C0G) (Figure 4). Interactions of each gene were counted and genes with nine common hub genes, i.e., genes with a maximum number of edges, were pinpointed (Table 7). Performing biological term enrichment analyses on those nine genes, biological processes related to cell cycle G2/M phase transition, histone phosphorylation and DNA integrity checkpoint were identified. The predominant KEGG pathway highlighted was cell cycle.

### 3.19. Other DEG Analyses

Comparisons involving early (X3μG-X0μG) (Table S23), late (X24μG-X0μG) (Table S24) and late vs. early (X24μG-X3μG) (Table S25) responses to X-ray under simulated μG had similar effects to early (X3G-X0G), late (X24G-X0G) and late vs. early (X24G-X3G) responses to X-ray under 1G. Comparisons concerning early (C3μG-C0μG) (Table S26), late (C24μG-C0μG) (Table S27) and late vs. early (C24μG-C3μG) (Table S28) responses to C-ion radiation under simulated μG had similar effects to early (C3G-C0G), late (C24G-C0G) and late vs. early (C24G-C3G) responses to C-ion radiation under 1G.

## 4. Discussion

In this space radiation-related work and through extensive transcriptomic analysis, using two distinct radiations (X-rays or C-ions), several key and original findings were retrieved. First, comparisons between 24 h post-irradiation vs. sham irradiation (0 h) identified more DEGs than comparisons between 3 h post-radiation vs. 0 h. More specifically, and based on biological term enrichments, processes related to response to DNA damage stimulus were identified 3 h post-irradiation, and suppressed 24 h post-irradiation [49]. Signal transduction by p53 and its downstream gene *CDKN1A* (p21) class mediator were also identified mainly in early response, as previously shown [50]. *CDKN1A* was found to be over-expressed 3 and 24 h after irradiation with 1 Gy of X-ray and 1 Gy of C-ion, as it was previously found [1]. FOXO4 (Forkhead Box O4) was identified as a transcription factor inducing genes in response to irradiation [4]. FOXOs are transcription factors that play a crucial role in cell fate decision and are involved in the promotion of apoptosis [51]. *CDKN1A*, *MDM2*, *PURPL*, *PTCHD4*, *TP53INP1*, *PAPPA* and *BTG2* were found to be up-regulated post-radiation in 1G and simulated μG condition, having the lowest *p*-values, as well as the highest Log_2_FoldChanges. *MDM2* was found to be over-expressed after irradiation with 1 Gy of X-ray and 1 Gy of C-ion, as previously found [1]. *TP53INP1* was found to be up-regulated in human irradiated fibroblasts and is associated with the regulation of apoptosis [52,53]. *MKI67* and *CCNB1* had a lower expression in response to radiation, especially 24 h post-irradiation in 1G or simulated μG conditions [1]. Histone-clustered genes (*H2AC13*, *H3C2*, *H3C10*, *H3C8* and *H4C3*) were found to be down-regulated genes post-radiation in 1G and simulated μG condition, as radiation suppressed the expression of various histone-clustered genes [54].

**Figure 5:**
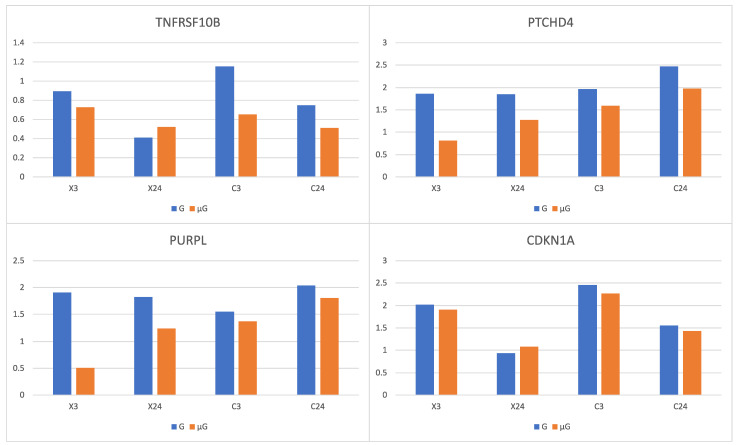
Log_2_ fold changes of up-regulated genes 3 h (X3) or 24 h (X24) after X-ray exposure vs. sham irradiation and 3 h (C3) or 24 h (C24) after C ion exposure vs. sham irradiation under 1G (G) (in blue) or simulated microgravity (μG) (in orange).

At a second level, the combined effect of C-ion or X-ray and simulated μG resulted in the up-regulation of *TNFRSF10B*, *PTCHD4* and *PURPL*. Their expression increased with irradiation alone, but their expression increase was lower when irradiation was combined with simulated μG than when it was combined with 1G. The expression of *CDKN1A* was found to behave in a similar way, but its lower increase was less statistically significant (Figure 5). It has been reported that the combined effect of C-ion radiation and simulated μG results in a lower increase of the expression of *CDKN1A*, compared to the effect of C-ion radiation treatment alone [1]. The expression of *TNFRSF10B* significantly increased in irradiated germline stem cells (GSCs) [55]. TNFSF10-TNFRSF10B pathway was found to be involved in radiation-induced apoptosis. The combined effect of radiation and simulated μG, may reduce the role of the TNFSF10-TNFRSF10B pathway that is crucial in the regulation of response to radiation, suppressing the process of apoptosis, meaning unrepaired cells not to undergo apoptosis, potentially causing the duplication of damaged cells.

**Figure 6:**
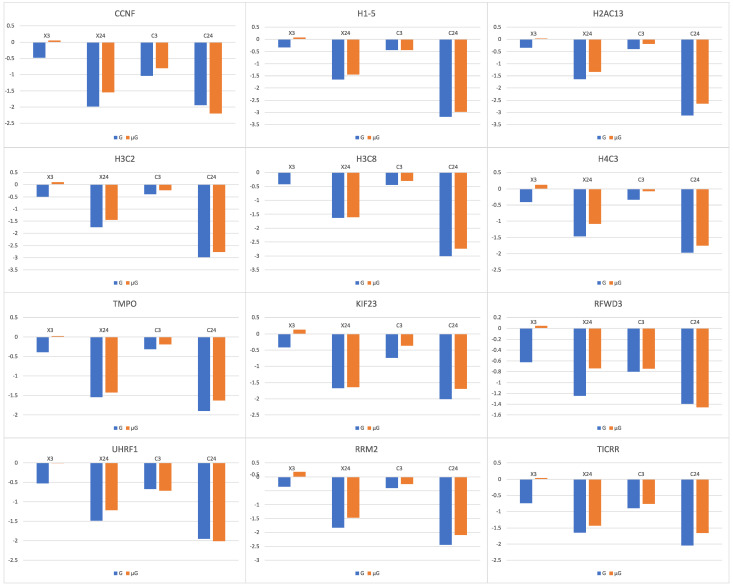
Log_2_ fold changes of down-regulated genes 3 h (X3) or 24 h (X24) after X-ray exposure vs. sham irradiation and 3 h (C3) or 24 h (C24) after C ion exposure vs. sham irradiation under 1G (G) (in blue) or simulated microgravity (μG) (in orange).

*PURPL* is a long non-coding RNA (lncRNA) [56]. *PURPL* has been reported to be up-regulated after DNA damage [44]. *PURPL* expression is anti-correlated with that of *TP53*. It was suggested that its anti-apoptotic action is due to its regulation of the *TP53* gene [56]. It was found that in the 12 genes (*CCNF*, *H1-5*, *H2AC13*, *H3C2*, *H3C8*, *H4C3*, *TMPO*, *KIF23*, *RFWD3*, *UHRF1*, *RRM2*, *TICRR*) whose expression was decreased with irradiation alone, simulated μG in combination with irradiation resulted in a lower gene expression decrease (Figure 6). The lowered transcriptional response to irradiation under simulated μG of genes that are related to cell apoptosis and DNA damage repair may explain the observed increase in chromosome aberrations in cells that were exposed simultaneously to radiation and simulated μG [2].

On a parallel direction of application of our accumulated data presented, a significant number of genes found to be affected by the combined effect of microgravity and radiation, are related to the following pathways: apoptosis, DNA damage response and repair, and cell cycle. All these pathways can be considered as potential drug targets for resistant tumors. Apoptosis-related genes *BLOC1S2*, *EDA2R*, *TP53INP1*, *MDM2*, *CDKN1A*, *FAS*, *BCL2L1*, repair-related genes *BRCA1*, *POLQ*, *BLM*, *H2AFX* or cell cycle-related genes *MKI67*, *CDT1*, *CDC6*, *MSH6*, *TERT* and other genes highly impacted by the microgravity environment open the possibility for targeting by microgravity conditions. Altered cancer cell gene expression after the application of microgravity can be used as a roadmap for the fight against cancer even without any other drug or chemical treatment [57]. For example, the down-regulation of cell growth or DNA repair genes can automatically sensitize cells to chemo- or radiotherapy. Another option would be to use a combination of microgravity with immunotherapy drugs. Last but not least, the results showing the ability of microgravity to especially impact cancer stem cells [15] underlines its possible use as a feasible tool in cancer therapy.

Comparing sham-irradiated cells under simulated μG and 1G, enrichment analyses in statistically significant down-regulated genes, highlighted terms related to the response to oxygen levels, muscle contraction and regulation of blood circulation. Based on their log_2_ fold changes, *TTN* and *MSTN* were identified as the most prominent down-regulated genes. *TTN* encodes for the lengthiest human protein, Titin, which controls sarcomere elasticity and contraction and is linked to the development of muscle atrophy [58]. As μG induces skeletal muscle atrophy [59,60], *TTN* under-expression under μG could be responsible for muscle mass loss in space flights. It has been found that an effect of μG is also the reduced human ventilatory response to hypoxia [61].

Biological processes related to the defense response (immune effector process and type I interferon signaling pathway) were highlighted in genes that were only differentially expressed under C-ion (high-LET) radiation but not in genes that were differentially expressed under X-ray (low-LET) radiation. Type I interferons are components of the early immune response. Strong associations between response to radiation and immune system and inflammation have been suggested in the past [62,63]. Although DNA repair was identified as an over-represented term in both aforementioned gene subsets, DDR terms such as double-strand break repair, double-strand break repair via the less-accurate non-homologous end joining (NHEJ) and non-recombinational repair were found exclusively in the genes that were differentially expressed under high-LET radiation [64]. The increased complexity of damage is often associated with a lethality increase compared to low-LET radiations [2]. Based also on our data for X-ray and C-ion-irradiated human G2-phase cells, it is suggested that classical NHEJ will make an initial attempt to repair the DSBs [65,66]. Examining up-regulated genes due to X-ray (low-LET) radiation, biological processes related to angiogenesis were discovered. Late down-regulated genes were found to be related to DNA damage repair, as it was previously shown [67]. Last but not least, relating to the biological effects of IR, the generation of oxidative stress is expected [68]. The lysyl oxidase (LOX) gene family contains five members: *LOX*; *LOXL1*; *LOXL2*; *LOXL3*; and *LOXL4* [69], all of which were found over-expressed as a response to radiation according to our results [70,71]. Hydrogen peroxide is a side product of this catalytic reaction. LOX proteins are expressed in fibroblasts [72]. Aberrant expression is involved in tumor invasion and metastasis [73]. Thus, lysyl oxidases provide targets for pharmacological and therapeutic intervention. Another cytochrome c oxidase subunit which is up-regulated in our experiments is cytochrome c oxidase subunit 7C (*COX7C*). Cytochrome c oxidase is related to the regulation of oxidative phosphorylation [74]. Other oxidases that we discovered to be over-expressed due to IR include Acyl-CoA oxidase 2 (*ACOX2*), Aldehyde oxidase 1 (*AOX1*) and Quiescin sulfhydryl oxidase 1 (*QSOX1*), all involved in the regulation of reactive oxygen species (ROS) homeostasis [75,76,77,78,79,80,81,82,83]. In addition, Glutathione peroxidase 1 (*GPX1*), a major antioxidant enzyme [84], was also found to be over-expressed as a response to IR in our analysis.

Conclusively, as key gene signatures, we have identified that *PLK1*, *BRCA1*, *CCNB1*, *AURKB*, *CDK1*, *CHEK1*, *RAD51*, *CCNA2* and *TOP2A* are hub genes in protein interaction networks for DEGs from the comparison between X-ray or C-ion-irradiated cells collected 24 h post-irradiation and sham-irradiated ones under 1G condition. Also, *BRCA1* and *RAD51* are associated with damage repair of DNA breaks. We note that *PLK1*, *CCNB1*, *AURKB*, *CDK1*, *CHEK1*, *CCNA2* and *TOP2A* play a critical role in the process of mitosis.

Our current findings suggest that human cells exposed to microgravity may significantly change their response to a genuine stressor like radiation. Alterations in biological responses to space-related radiations are known and the challenge that we aimed to address in this study was to bring into surface key processes impacted by the specific types of radiations and irradiation methodologies probing the exposure of human cells to space radiations. Therefore, the field is open for the use of these results in the development of new tools and methodologies to overcome tumor resistance beyond the current use in space missions.

## Figures and Tables

**Table 1:** Names and combinations of the type of radiation (C-ion or X-ray), collection time points (0 (sham irradiated), 3 or 24 h) and gravity (1G or simulated μG) for the 12 conditions studied.

Name	Irradiation		Maintenance Time			Gravity	
	C-ion	X-ray	0 h	3 h	24 h	1G	Simulated μG
C0G	−	−	+	−	−	+	−
C0μG	−	−	+	−	−	−	+
C3G	+	−	−	+	−	+	−
C3μG	+	−	−	+	−	−	+
C24G	+	−	−	−	+	+	−
C24μG	+	−	−	−	+	−	+
X0G	−	−	+	−	−	+	−
X0μG	−	−	+	−	−	−	+
X3G	−	+	−	+	−	+	−
X3μG	−	+	−	+	−	−	+
X24G	−	+	−	−	+	+	−
X24μG	−	+	−	−	+	−	+

**Table 2:** Comparisons between biological conditions (Table 1) that were performed in DEG analyses.

Comparisons	Description
X3G-X0G	Early response to X-ray under 1G
X3μG-X0μG	Early response to X-ray under μG
X0μG-X0G	Response to μG in sham-X-ray-irradiated cells
Χ3μG-X3G	Response to μG in cells collected 3 h after X-ray irradiation
(X3μG-X0μG)-(Χ3G-X0G)	Interaction between early response to X-ray and response to μG
X24G-X0G	Late response to X-ray under 1G
X24μG-X0μG	Late response to X-ray under μG
Χ24μG-X24G	Response to μG in cells collected 24 h after X-ray irradiation
(X24μG-X0μG)-(Χ24G-X0G)	Interaction between late response to X-ray and response to μG
X24G-X3G	Late vs. early response to X-ray under 1G
X24μG-X3μG	Late vs. early response to X-ray under μG
(X24μG-X3μG)-(Χ24G-X3G)	Interaction between late vs. early response to X-ray and response to μG
X3μG-X0G	Early response to X-ray irradiation and μG combined effect
X24μG-X0G	Late response to X-ray irradiation and μG combined effect
C3G-C0G	Early response to C-ion under 1G
C3μG-C0μG	Early response to C-ion under μG
C0μG-C0G	Response to μG in sham-C-ion-irradiated cells
C3μG-C3G	Response to μG in cells collected 3 h after C-ion irradiation
(C3μG-C0μG)-(C3G-C0G)	Interaction between early response to C-ion and response to μG
C24G-C0G	Late response to C-ion under 1G
C24μG-C0μG	Late response to C-ion under μG
C24μG-C24G	Response to μG in cells collected 24 h after C-ion irradiation
(C24μG-C0μG)-(C24G-C0G)	Interaction between late response to C-ion and response to μG
C24G-C3G	Late vs. early response to C-ion under 1G
C24μG-C3μG	Late vs. early response to C-ion under μG
(C24μG-C3μG)-(C24G-C3G)	Interaction between late vs. early response to C-ion and response to μG
C3μG-C0G	Early response to C-ion irradiation and μG combined effect
C24μG-C0G	Late response to C-ion irradiation and μG combined effect

**Table 3:** Common up-regulated genes between early- and late-response genes to X-ray and C-ion radiation under 1G.

Ensembl Gene ID	Gene Name	Description
ENSG00000136542	*GALNT5*	Polypeptide n-acetylgalactosaminyltransferase 5
ENSG00000196576	*PLXNB2*	Plexin b2
ENSG00000134574	*DDB2*	Damage specific dna binding protein 2
ENSG00000131080	*EDA2R*	Ectodysplasin a2 receptor
ENSG00000163071	*SPATA18*	Spermatogenesis associated 18
ENSG00000173846	*PLK3*	Polo like kinase
ENSG00000135679	*MDM2*	Mdm2 proto-oncogene
ENSG00000244509	*APOBEC3C*	Apolipoprotein b mrna editing enzyme catalytic subunit 3c
ENSG00000120889	*TNFRSF10B*	Tnf receptor superfamily member 10b
ENSG00000048392	*RRM2B*	Ribonucleotide reductase regulatory tp53 inducible subunit m2b
ENSG00000127241	*MASP1*	Mbl associated serine protease 1
ENSG00000182752	*PAPPA*	Pappalysin 1
ENSG00000172667	*ZMAT3*	Zinc finger matrin-type 3
ENSG00000244694	*PTCHD4*	Patched domain containing 4
ENSG00000174307	*PHLDA3*	Pleckstrin homology like domain family a member 3
ENSG00000171444	*MCC*	Mcc regulator of wnt signaling pathway
ENSG00000124762	*CDKN1A*	Cyclin dependent kinase inhibitor 1a
ENSG00000250337	*PURPL*	P53 up regulated regulator of p53
ENSG00000164938	*TP53INP1*	Tumor protein p53 inducible nuclear protein 1
ENSG00000132274	*TRIM22*	Tripartite motif containing
ENSG00000080546	*SESN1*	Sestrin 1
ENSG00000159388	*BTG2*	Btg anti-proliferation factor 2
ENSG00000164125	*GASK1B*	Golgi associated kinase 1b
ENSG00000154767	*XPC*	Xpc complex subunit, dna damage recognition and repair factor
ENSG00000167196	*FBXO22*	F-box protein 22
ENSG00000161513	*FDXR*	Ferredoxin reductase

**Table 4:** Common down-regulated genes between early- and late-response genes to X-ray and C-ion radiation under 1G.

Ensembl Gene ID	Gene Name	Description
ENSG00000156802	*ATAD2*	ATPase family AAA domain containing 2
ENSG00000175305	*CCNE2*	Cyclin E2
ENSG00000162063	*CCNF*	Cyclin F
ENSG00000094804	*CDC6*	Cell division cycle 6
ENSG00000167513	*CDT1*	Chromatin licensing and DNA replication factor 1
ENSG00000106462	*EZH2*	Enhancer of zeste 2 polycomb repressive complex 2 subunit
ENSG00000189057	*FAM111B*	FAM111 trypsin like peptidase B
ENSG00000184357	*H1-5*	H1.5 linker histone, cluster member
ENSG00000196747	*H2AC13*	H2A clustered histone 13
ENSG00000286522	*H3C2*	H3 clustered histone 2
ENSG00000278828	*H3C10*	H3 clustered histone 10
ENSG00000273983	*H3C8*	H3 clustered histone 8
ENSG00000197061	*H4C3*	H4 clustered histone 3
ENSG00000137807	*KIF23*	Kinesin family member 23
ENSG00000065328	*MCM10*	Minichromosome maintenance 10 replication initiation factor
ENSG00000112118	*MCM3*	Minichromosome maintenance complex component 3
ENSG00000104738	*MCM4*	Minichromosome maintenance complex component 4
ENSG00000116062	*MSH6*	Muts homolog 6
ENSG00000085840	*ORC1*	Origin recognition complex subunit 1
ENSG00000161800	*RACGAP1*	Rac gtpase activating protein 1
ENSG00000168411	*RFWD3*	Ring finger and WD repeat domain 3
ENSG00000171848	*RRM2*	Ribonucleotide reductase regulatory subunit M2
ENSG00000140534	*TICRR*	TOPBP1 interacting checkpoint and replication regulator
ENSG00000120802	*TMPO*	Thymopoietin
ENSG00000276043	*UHRF1*	Ubiquitin like with PHD and ring finger domains 1
ENSG00000162607	*USP1*	Ubiquitin-specific peptidase 1
ENSG00000092470	*WDR76*	WD repeat domain 76

**Table 5:** Three up-regulated genes whose expression increased with irradiation alone, while simulated μG in combination with irradiation results in a lower gene expression increase.

Ensembl Gene ID	Gene Name	Description
ENSG00000120889	*TNFRSF10B*	TNF receptor superfamily member 10b
ENSG00000244694	*PTCHD4*	Patched domain containing 4
ENSG00000250337	*PURPL*	P53 up-regulated regulator of p53

**Table 6:** 12 down-regulated genes whose expression decreased with irradiation alone, while simulated μG in combination with irradiation results in a lower gene expression decrease.

Ensembl Gene ID	Gene Name	Description
ENSG00000162063	*CCNF*	Cyclin F
ENSG00000184357	*H1-5*	H1.5 linker histone, cluster member
ENSG00000196747	*H2AC13*	H2A clustered histone 13
ENSG00000286522	*H3C2*	H3 clustered histone 2
ENSG00000273983	*H3C8*	H3 clustered histone 8
ENSG00000197061	*H4C3*	H4 clustered histone 3
ENSG00000120802	*TMPO*	Thymopoietin
ENSG00000137807	*KIF23*	Kinesin family member 23
ENSG00000168411	*RFWD3*	Ring finger and WD repeat domain 3
ENSG00000276043	*UHRF1*	Ubiquitin like with PHD and ring finger domains 1
ENSG00000171848	*RRM2*	Ribonucleotide reductase regulatory subunit M2
ENSG00000140534	*TICRR*	TOPBP1 interacting checkpoint and replication regulator

**Table 7:** Nine common hub DEGs from the comparisons between X-ray-irradiated cells collected 24 h post-irradiation and sham-irradiated ones under 1G, as well as between C-ion-irradiated cells collected 24 h post-irradiation and sham-irradiated ones under 1G.

Ensembl Gene ID	Gene Name	Description
ENSG00000166851	*PLK1*	Polo-like kinase 1
ENSG00000012048	*BRCA1*	BRCA1 DNA repair associated
ENSG00000134057	*CCNB1*	cyclin B1
ENSG00000178999	*AURKB*	aurora kinase B
ENSG00000170312	*CDK1*	cyclin dependent kinase 1
ENSG00000149554	*CHEK1*	checkpoint kinase 1
ENSG00000051180	*RAD51*	RAD51 recombinase
ENSG00000145386	*CCNA2*	cyclin A2
ENSG00000131747	*TOP2A*	DNA topoisomerase II alpha

## Data Availability

The raw data supporting the conclusions of this article will be made available by the authors on request.

## Supplementary Materials

The following supporting information can be downloaded at: https://www.mdpi.com/article/10.3390/biom14010088/s1, Table S1: X3G-X0G. Lists of DEGs and enriched biological terms in early response to X-ray under 1G; Table S2: X24G-X0G. Lists of DEGs and enriched biological terms in late response to X-ray under 1G; Table S3: X24G-X3G. Lists of DEGs and enriched biological terms in late vs. early response to X-ray under 1G; Table S4: C3G-C0G. Lists of DEGs and enriched biological terms in early response to C-ion radiation under 1G; Table S5: C24G-C0G. Lists of DEGs and enriched biological terms in late response to C-ion radiation under 1G; Table S6: C24G-C3G. Lists of DEGs and enriched biological terms in late vs. early response to C-ion radiation under 1G; Table S7: X0μG-X0G. Lists of DEGs and enriched biological terms in response to simulated μG in sham-X-ray-irradiated cells; Table S8: C0μG-C0G. Lists of DEGs and enriched biological terms in response to simulated μG in sham-C-ion-irradiated cells; Table S9: C3μG-C3G. Lists of DEGs and enriched biological terms in response to simulated μG in cells collected 3 h after C-ion irradiation; Table S10: X3μG-X3G. Lists of DEGs and enriched biological terms in response to simulated μG in cells collected 3 h after X-ray irradiation; Table S11: X24μG-X24G. Lists of DEGs in response to simulated μG in cells collected 24 h after X ray-irradiation; Table S12: C24μG-C24G. Lists of DEGs and enriched biological terms in response to simulated μG in cells collected 24 h after C-ion irradiation; Table S13: X3μG-X0G. Lists of DEGs and enriched biological terms in early response to X-ray irradiation and simulated μG combined effect; Table S14: X24μG-X0G. Lists of DEGs and enriched biological terms in late response to X-ray irradiation and simulated μG combined effect; Table S15: C3μG-C0G. Lists of DEGs and enriched biological terms in early response to C-ion irradiation and simulated μG combined effect; Table S16: C24μG-C0G. Lists of DEGs and enriched biological terms in late response to C-ion irradiation and simulated μG combined effect; Table S17: (X3μG-X0μG)-(Χ3G-X0G). Lists of DEGs in response to the interaction between early response to X-ray and response to simulated μG; Table S18: (X24μG-X0μG)-(Χ24G-X0G). Lists of DEGs in response to the interaction between late response to X-ray and response to simulated μG; Table S19: (C3μG-C0μG)-(C3G-C0G). Lists of DEGs in response to the interaction between early response to C-ion and response to simulated μG; Table S20: (C24μG-C0μG)-(C24G-C0G). Lists of DEGs in response to the interaction between late response to C-ion and response to simulated μG; Table S21: (X24μG-X3μG)-(Χ24G-X3G). Lists of DEGs in response to the interaction between late vs. early response to X-ray and response to simulated μG; Table S22: (C24μG-C3μG)-(C24G-C3G). Lists of DEGs in response to the interaction between late vs. early response to C-ion and response to simulated μG; Table S23: X3μG-X0μG. Lists of DEGs and enriched biological terms in early response to X-ray under simulated μG; Table S24: X24μG-X0μG. Lists of DEGs and enriched biological terms in late response to X-ray under simulated μG; Table S25: X24μG-X3μG. Lists of DEGs and enriched biological terms in late vs. early response to X-ray under simulated μG; Table S26: C3μG-C0μG. Lists of DEGs and enriched biological terms in early response to C-ion under simulated μG; Table S27: C24μG-C0μG. Lists of DEGs and enriched biological terms in late response to C-ion under simulated μG; Table S28: C24μG-C3μG. Lists of DEGs and enriched biological terms in late vs. early response to C-ion under simulated μG.
